# Fluorescence-Based Measurement of Workpiece Geometry and Temperature in Laser Chemical Machining

**DOI:** 10.1007/s41871-025-00257-w

**Published:** 2025-06-30

**Authors:** Claudia Niehaves, Andreas Tausendfreund, Yasmine Bouraoui, Yang Lu, Tim Radel, Andreas Fischer

**Affiliations:** 1 Bremen Institute for Metrology, Automation and Quality Science, University of Bremen, Linzer Str. 13, 28359 Bremen, Germany; 2 BIAS - Bremer Institut für angewandte Strahltechnik, Klagenfurter Str. 5, 28359 Bremen, Germany

**Keywords:** Laser chemical machining, In situ measurement, Geometry, Temperature

## Abstract

Laser chemical machining (LCM) is a gentle metal removal technique with micrometer resolution. LCM involves laser-driven surface heating of the workpiece, which is subjected to a flowing acid bath, locally inducing a chemical dissolution reaction. To ensure a high machining quality, the laser power is intentionally limited to avoid disturbances in material removal presumably caused by the shielding effect of boiling bubbles. To achieve both an increased removal rate and a high removal quality, the current understanding of surface removal mechanisms must be fundamentally expanded. Therefore, to create the basis of near-process quality control in the future, a near-process measurement approach is needed for the machined workpiece geometry inside the machine and the temperature in the process fluid as an important process quantity. This study introduces a fluorescence-based measurement approach capable of assessing both quantities in-situ. An experimental feasibility study demonstrated the robustness of the approach in measuring the three-dimensional geometry of a structure produced by LCM, even in the presence of streaming air bubbles in the optical path, thereby validating its near-process capability. However, systematic measurement errors, such as edge artifacts, were observed in the geometry measurements, indicating the need for a revision of the signal model. In addition, precise temperature measurements of the electrolyte solution within the LCM environment were achieved, with a random error of $1\,^{\circ }\hbox {C}$ and a systematic error of $1.4\,^{\circ }\hbox {C}$.

## Highlights

1. A scanning confocal fluorescence microscope is realized to enable in-situ measurements in laser chemical machining.
2. The 2D geometry of the workpiece is measurable, even in case of process-caused gas bubbles, by evaluating the liquid’s fluorescence intensity.
3. The temperature of the electrolyte is measurable with the identical optical setup by additionally evaluating the fluorescence lifetime.

## Introduction

### Motivation

Alternative laser-based machining methods, such as laser chemical machining (LCM), are developed with the potential to process hard-to-machine materials, such as titanium and Inconel, far below the melting temperature. In this way, issues associated with the liquid phase, such as cracking, are avoided. LCM combines the benefit of laser processing, i.e., a locally precise, flexible, and wear-free energy input, with the gentle and smooth processing of electrochemical machining. LCM applies to metals with a passivation layer on the surface that protects against corrosion in an etching electrolyte solution. By laser-driven surface heating, a chemical dissolution reaction is activated locally at the interface between the electrolyte and the workpiece, resulting in the selective removal of the passivation layer. With LCM, a high surface quality with roughness values less than $100\, \textrm{nm}$ can be achieved [1, 2], and the generation of complex removal profiles by laser power modulation was recently demonstrated [3]. However, the processing window of LCM is restricted in terms of temperature and, therefore, laser intensity. Outside of this processing window, disturbances in material removal occur presumably because of the shielding effect of emerging boiling bubbles in the electrolyte fluid [4]. Current (blurred) high-speed videos obtained by Lu et al. [5] show permanent bubble generation during LCM, which can have a dramatic, unpredictable dynamic at higher laser powers. To avoid severe gas bubbles, the laser power is limited at a given beam diameter, resulting in a limited removal rate of typically $10^{-3}\, \mathrm {mm^3/min}$ for LCM [6]. This removal rate is significantly slower than that of other micromanufacturing processes, such as laser ablation with ultrafast lasers operating at high pulse repetition rates, which can achieve removal rates of more than $1\, \mathrm {mm^3/min}$ [7, 8]. To increase the removal rate, the current understanding of surface removal mechanisms must be fundamentally expanded, which necessitates near-process measurements of the removal geometry and the electrolyte temperature under the in-situ process conditions of LCM.

### State of the Art

To date, no near-process measurement techniques for LCM exist. Typically, the machined workpiece geometry is inspected ex-situ after fabrication using different techniques, such as confocal scanning microscopy [3] or white light interferometry (WLI). The required spatial resolution for LCM is in the single-digit micrometer range.

To characterize laser chemically fabricated structures in-situ, a noncontact inspection method of the microtopography is required because of the difficult-to-access conditions inside the etching cell. Therefore, an optical measurement method is the preferred choice. However, the refractive index variations due to emerging bubbles or temperature gradients exclude interferometric methods that are based on the determination of phase differences. Conversely, laser chemically produced microcavities with a steepness of greater than $75\,^{\circ }$ lead to artifacts in optical systems that rely on surface-reflected light detection, such as confocal microscopy [9].

In preliminary ex-situ investigations, an indirect measurement method using confocal fluorescence microscopy has been proven to be a promising approach for an in-situ-capable geometry measurement technique [10]. The workpiece geometry is determined indirectly from the boundary layer of the fluid covering the workpiece surface by detecting the fluorescent light emitted by the fluid. The fluorescence property of the process fluid can be inherent (as for lubricants) or generated by a fluorescent dye. Different studies have demonstrated the use of laser-induced fluorescence to sense surfaces or surface-related quantities in machining environments. For instance, the profile of a cutting tool has been characterized [11, 12] or the lubricant thickness of the tool–workpiece interface during indentation has been measured this way [13]. Because these studies focused on fluorescent fluid layers with thicknesses in the micrometer range or smaller, the model-based signal evaluation was extended to cope with the millimeter-thick electrolyte layer of the LCM process [10]. However, a near-process on-machine measurement has not been demonstrated so far. Furthermore, an extension of 1D profile measurements toward scanning 2D geometry measurements is pending. Finally, how to measure the electrolyte temperature in addition to the workpiece geometry needs to be clarified, ideally with a common-path approach, because of the restricted optical access. In LCM, the electrolyte flows constantly, contributing to heat transfer; however, the resulting temperature distribution remains unclear. To integrate local temperature measurements into the existing confocal microscope setup, the temperature dependence of the emission of the fluorescent dye is utilized. The fluorescence properties affected by temperature include the emitted spectrum, intensity, and fluorescence lifetime. In Ref. [14], for example, temperature measurements during polymer processing were realized by monitoring the spectral changes of a fluorescent dye incorporated into the polymer resin (in combination with a confocal optical setup). However, fluorescence spectra are also sensitive to fluctuations in the lighting conditions or the dye concentrations, which make quantitative temperature measurement difficult. In contrast to any ratiometric evaluation of the fluorescence intensity, the fluorescence lifetime, which is measured by tracking the fluorescence decay over time, remains largely unaffected by these illumination conditions. Although the fluorescence lifetime can be measured in the time or frequency domain [15], the measurement of the fluorescence lifetime in the time domain via time-correlated single-photon counting (TCSPC) yields the best lifetime accuracy [16]. To this end, a pulsed excitation source is used, and individual photons (emitted by the dye) are detected by a fast single-photon detector. To date, fluid temperature measurement via TCSPC is widely used in biological or biomedical in-situ investigations, e.g., in cellular environments [17, 18], and frequently applied in fluid mechanic experiments, e.g., for droplet temperature measurements [19]. However, whether temperature measurement using the fluorescence lifetime (via TCSPC) is possible in the etching fluid and whether the approach can be combined with the proposed indirect geometry measurement as an in-situ monitoring tool for LCM remain open questions.

### Aim and Outline

This study aims to investigate the feasibility of in-situ fluorescence-based measurements of the three-dimensional (3D) removal geometry and the fluid temperature in the LCM process environment, i.e., inside the etching cell, with a fluid flow. For this purpose, a scanning confocal fluorescence microscope setup is installed on the LCM machine. To assess the fundamental 3D geometry measurement capabilities of the on-machine setup, first, the workpiece is measured in a Petri dish with Rhodamine B (RhB) dissolved in water, whereas the reference is provided by ex-situ measurements with a white light interferometer. Then, the feasibility of measuring the workpiece geometry in the etching cell is clarified. In addition, the influence of a stream of gas bubbles in the optical path, which is of particular relevance for the near-process or in-process capability of the measurement approach, is investigated. Finally, fluid temperature measurements are conducted by evaluating the fluorescence lifetime, except for the change of the detector, with the same optical measurement setup. The measurement principles for detecting the surface geometry and temperature via confocal fluorescence microscopy, including the implemented signal processing, are described in Sect. 2. The experimental setup of the measurement system on the LCM machine is explained in Sect. 3. The experimental results are presented and discussed in Sect. 4. The key findings, complemented by an outlook on future research works, are summarized in Sect. 5.

## Measurement Principle

### Workpiece Geometry

**Fig. 1:**
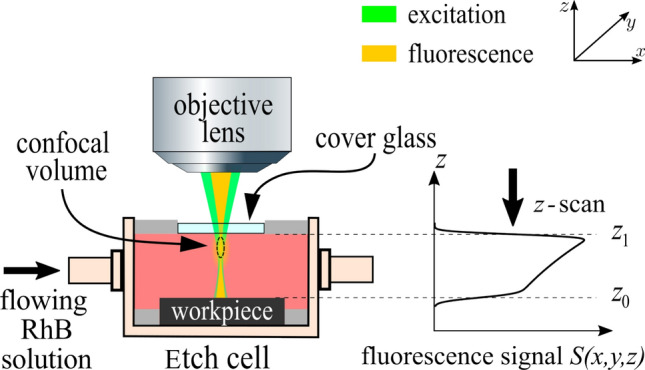
Indirect geometry measurement by fluorescence and confocal microscopy

Liquids involved in the manufacturing environment (e.g., phosphoric acid in the LCM process) can be enriched by a fluorescent dye and excited to fluoresce by a focused laser source. This generated fluorescence emission in the fluid covering the workpiece is the basis for the in-situ measurement principle of the electrolyte temperature and surface geometry of the metallic sample. For the detection of fluorescent light, a confocal microscope system is chosen. This microscopy technique is characterized by a pinhole that is arranged conjugate to the focal plane (confocal), which hinders the detection of out-of-focus emissions and provides the possibility of optical sectioning along the optical axis. A schematic representation of the measurement principle is depicted in Fig. 1. The spatial distribution of the detected fluorescent light in the so-called confocal volume is approximated by a 3D Gaussian function $I(\varvec{r},z)$, expressed as follows [20]:

1 $$\begin{aligned} {\begin{matrix} I(\varvec{r}, z) = & A \cdot \epsilon \cdot \exp \Big (- \frac{2}{\omega ^2}\Big (\varvec{r}^2 + \frac{z^2}{\kappa ^2}\Big ) \Big ), \\ & \text {with} \quad \varvec{r} = \left( \begin{array}{c} x \\ y \end{array}\right) , \end{matrix}} \end{aligned}$$

where the width of the distribution is $\omega$ in the *x*- and *y*-directions and $\omega \cdot \kappa$ in the *z*-direction. Hence, the factor $\kappa$ is the aspect ratio of the lateral and axial dimensions of the confocal volume. The fluorophore concentration is taken into account by the attenuation coefficient $\epsilon$, and the excitation power and fluorophore quantum yield are taken into account by the parameter *A*. By performing a depth scan, i.e., a movement of the confocal volume (illumination and detection) in the *z*-direction until reaching the surface of the specimen, a characteristic fluorescence signal *S*(*z*) is detected, see Fig. 1(right), from which the surface position $z_0$ is derived. The scanned intensity signal *S*(*z*) of the fluorescent light is detected inside the fluid layer, i.e., for the *z* values between the liquid surface at $z_1$ and the surface position $z_0$. At the boundary $z_0$, the signal decrease is distributed over a certain width, which can be described mathematically by a convolution of the confocal volume $I(\varvec{r}, z)$ and the weighting function $\eta (z)$, expressed as follows:

2 $$\begin{aligned}  S(z) = \,\,& \eta (z) *\int I(\varvec{r},z)\, \textrm{d}\varvec{r},\\ \text {with} \quad & \eta (z) = {\left\{ \begin{array}{ll} {\text{e}}^{\epsilon (z-z_1)} \quad {z_0} \le {z} \le {z_1} \\ 0 \quad \quad \quad \,\, \text {otherwise,}\end{array}\right. }  \end{aligned}$$

where $\eta (z)$ is the exponential decrease in intensity according to Lambert–Beer’s law of light attenuation due to absorption. Notably, any effect of surface reflection is currently excluded from the model function. Furthermore, for the sake of simplicity, this model function assumes a surface that is perpendicular to the optical axis, i.e., an oblique or curved truncation of the confocal volume caused by an inclined or highly curved surface is not part of the model. The evaluation of the convolution integral results in the following expression:

3 $$\begin{aligned}  S(z) = \,\,& S_0 \bigg (\textrm{erf}\Big (\frac{z-z_0}{2 \Xi } + \epsilon \Xi \Big ) \\ & - \textrm{erf}\Big ( \frac{z-z_1}{2 \Xi } + \epsilon \Xi \Big )\bigg ) \,{\text{e}}^{\epsilon (z-z_1)},  \end{aligned}$$

where $\Xi = \frac{1}{4} \sqrt{2} \kappa \omega$ is a parameter describing the axial extent of the confocal volume. Then, the surface position $z_0$ is determined by nonlinear regression of the measured fluorescence intensity signal with the model function *S*(*z*) using the least squares method.

### Electrolyte Temperature

**Fig. 2:**
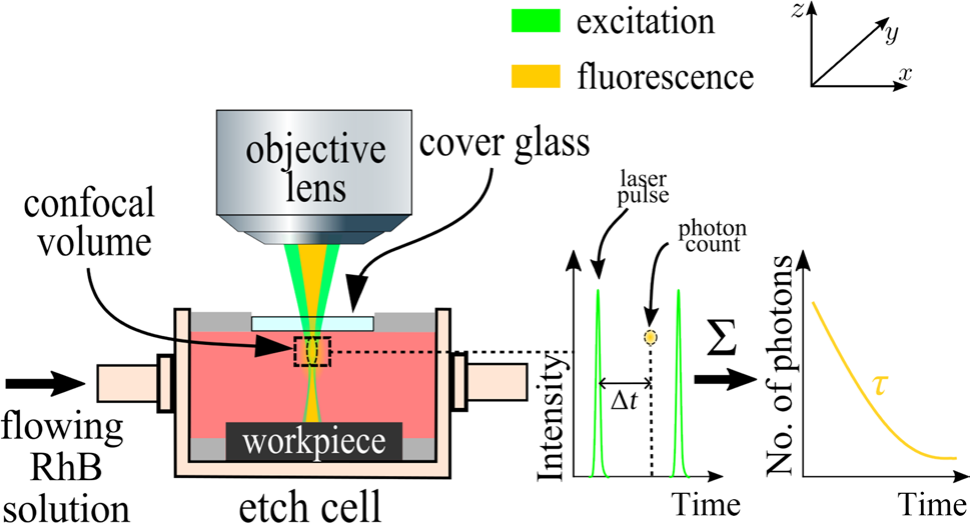
Temperature measurement based on the fluorescence lifetime (TCSPC principle)

The fluorescence lifetime is sensitive to the ambient conditions of the fluorophore, such as electrolyte temperature, enabling it to serve as a molecular probe of the process environment. When a population of fluorescent molecules is excited by an ultrashort laser pulse, the time-resolved fluorescence signal exhibits an exponential decay. A well-established method to determine the decay rate of the fluorescence signal is known as TCSPC. For this, a (fast) time-correlating counter is necessary as a stopwatch that measures the elapsed time between the excitation pulse and the subsequent detection of a photon event. To excite only a single photon per excitation cycle, i.e., to avoid any pile-up effect, the laser light intensity has to be sufficiently low. The measurement is repeated over many cycles of excitation pulses, and the collected time recordings $\Delta t$ are sorted into a histogram, see Fig. 2. For many fluorophores, the resulting histogram exhibits a multiexponential drop of photon counts that is modeled by the sums of the exponential functions, including more than a single decay time $\tau$. To derive the fluorescence lifetime $\tau$ of RhB dissolved in water, a biexponential fit with the fluorescence lifetimes $\tau _1$ and $\tau _2$ is reported and expressed as follows [19]:

4 $$\begin{aligned} \frac{S\left( t\right) }{S\left( t_{\textrm{max}}\right) }=a{\text{e}}^{-t/\tau _1}+\left( 1-a\right) {\text{e}}^{-t/\tau _2}, \end{aligned}$$

with $0<a <1$. Then, the average fluorescence lifetime $\tau$ is calculated using the following expression:

5 $$\begin{aligned} \tau =a\tau _1+\left( 1-a\right) \tau _2. \end{aligned}$$

Meanwhile, the relationship between the fluorescence lifetime and the sought-after temperature is determined by calibration.

## Experimental Setup

**Fig. 3:**
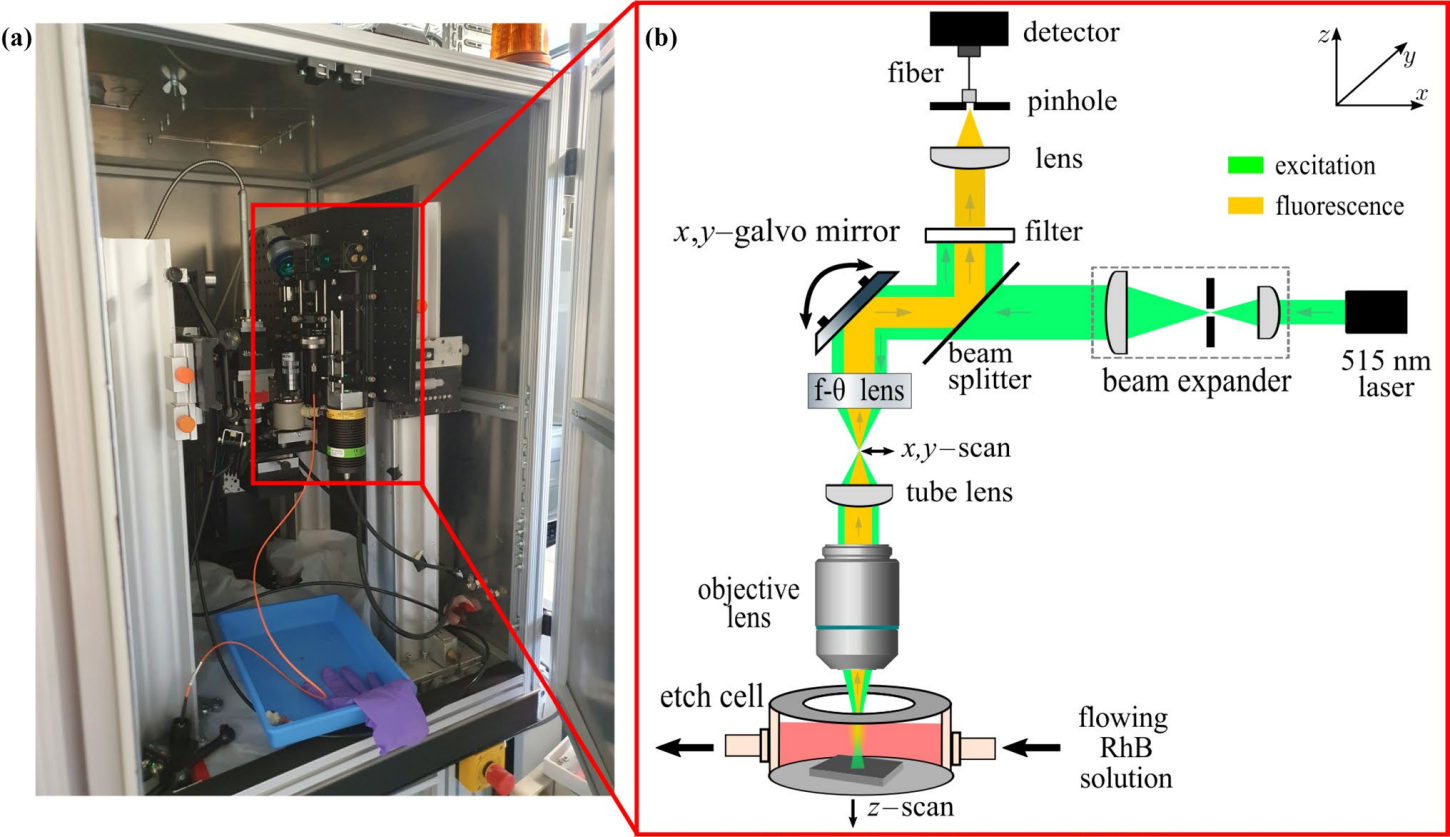
**a** Photography of the LCM plant. **b** Schematic of the scanning confocal fluorescence microscopy setup realized to be in-situ-capable

**Fig. 4:**
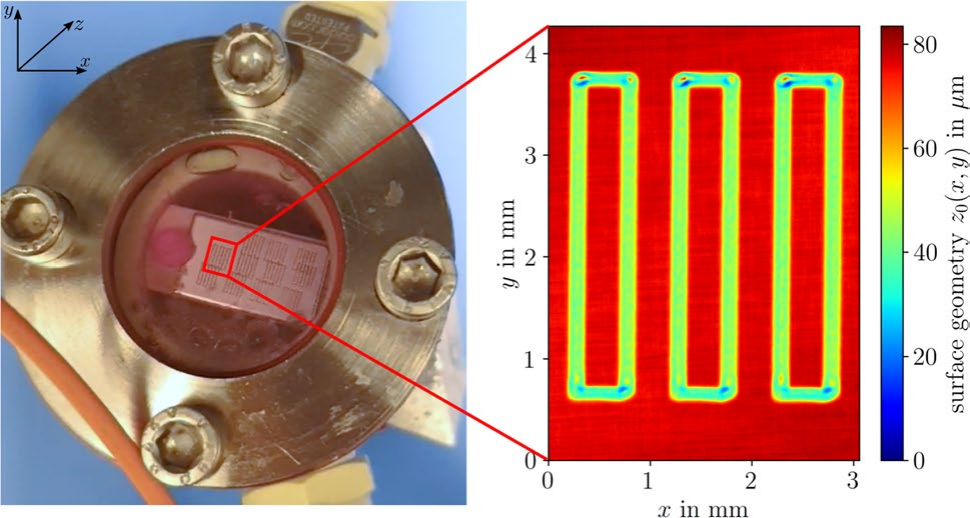
Left: Top view of the etching cell. Right: Reference measurement of the LCM structure by white light interferometry (WLI)

Figure 3 presents the measurement setup integrated into the LCM system. The practical measurement setup for measuring the geometry and near-surface temperature realized at the BIAS–Bremer Institut für Angewandte Strahltechnik is shown in Fig. 3a, with the processing chamber of the LCM system open. The optical system of the in-situ scanning confocal fluorescence microscope is shown schematically in Fig. 3b. The top view of the etching cell used in the LCM process is shown in Fig. 4. For processing, the workpiece is clamped inside the closed etching cell. The cell has a processing window that is covered by a 10-mm-thick glass plane. Between this plane and the sample is a gap with a width of 4 mm, where the etching medium is pumped through. Phosphoric acid (5 mol) is used as the etching medium. A typical volume flow rate for the LCM process is $300\, \mathrm {mL/min}$, which corresponds approximately to an estimated (mean) flow velocity of $30\, \mathrm {mm/s}$ inside the etching cell. As a fluorescent dye, RhB is dissolved in the etching medium. The etching cell is positioned on a motorized linear stage (Newport) that performs the movement in the *z*-direction, controlled by a motion controller (ESP302, Newport). A scan in the *z*-direction is required to determine the surface position by the indirect optical geometry measurement approach. A pulsed diode laser (LDH-P-C-520B, Picoquant) with a central wavelength of $515\, \textrm{nm}$ and a maximum power of $4\, \textrm{mW}$ is used as an excitation source. The laser was operated with a repetition rate of 40 MHz for the geometry measurements, controlled by a driver for picosecond pulses (PDL 800-D, Picoquant). The pulse width of the laser is less than 170 ps (FWHM). The laser light is expanded by a Keplerian beam expander, conducted through a beam splitter, and projected onto a two-axis galvo mirror. The mirror scans the illumination over the sample (*x*- and *y*-directions) with a scan frequency of 20 Hz. To this end, a sawtooth and a stair step voltage signal are supplied for the *x*- and *y*-axes, respectively. After completing a full lateral scan, the system advances incrementally along the *z*-axis. As a result, a measuring volume of typically $(x,y,z) = (0.5, 1.7, 4\, \textrm{mm})$ is covered by each measurement. The light is focused by an f-theta lens (LSM03-VIS, Thorlabs), which guarantees a flat image plane. After passing the collimating lens, the laser light illuminates the objective lens (Plan Apo 20X, $\#59$-878, Edmund Optics) that has a numerical aperture of 0.42 and a working distance of 20 mm. The objective focuses the light on the fluid in the etching cell, inducing the excitation of fluorescence. The diffusely emitted fluorescent light is recollected by the same objective lens and redirected to the beam splitter. A long-pass filter with a cut-on wavelength of $550\, \textrm{nm}$ separates the excitation light from the fluorescent light, which is focused on a pinhole with a diameter of 50 μm. For the geometry measurement, the average fluorescent light over many laser pulses is ultimately detected by an avalanche photodiode (APD440A2, Thorlabs). The pulse rate is so high that the laser can be regarded as a quasi-continuous wave source for the geometry measurements. An I/O controller board from National Instruments (NI USB-6621) samples the signal from the avalanche photodiode at a sampling rate of 100 kHz and records it on a computer together with the scanning signals. As a result, the detected fluorescent light is assigned to the scan path. For the measurement of the fluorescence lifetime, a laser pulse repetition rate of 20 MHz is chosen, and the avalanche photodiode is replaced by a single-photon sensitive photomultiplier tube (PMA-C 175-M, Picoquant). The output of the single-photon detector is captured by fast counting electronics (MultiHarp 150 4N, Picoquant) with 80 ps time resolution to record the time difference between the excitation pulse and the emission of the fluorescence photon.

## Results

### Geometry Measurement of LCM Sample

**Fig. 5:**
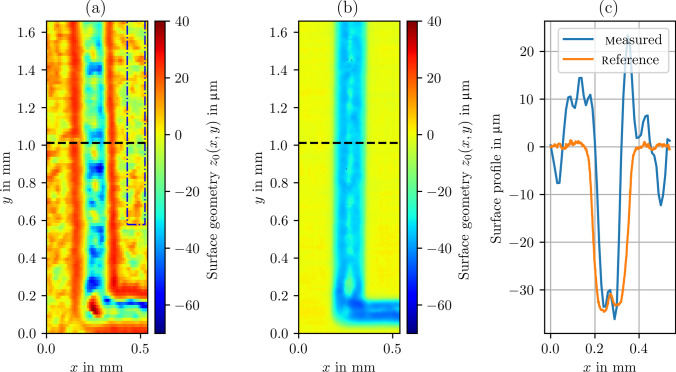
**a** Geometry measurement of the LCM structure in a petri dish. **b** WLI reference measurement. **c** Surface profiles extracted from **a** and **b** denoted by the dashed line

To examine and validate the 3D geometry measurement capability of the established in-situ confocal fluorescence microscope and the indirect measurement approach, a laser chemically machined surface was measured in a Petri dish. The Petri dish was filled with RhB dissolved in water (to avoid any interference by the electrolyte). The examined workpiece is a grade 1 titanium plate featuring parabolic-shaped removal cavities arranged in a rectangular pattern (cf. Fig. 4). The cavities have a width of approximately $200\, \mathrm {\mu m}$ and a depth of approximately $30\, \mathrm {\mu m}$. Using a raster scan, fluorescence is detected point by point above the cavity. The lateral scan was performed at discrete *z*-positions. To calibrate the *x*- and *y*-scales of the scan, the measurement was compared with the reference measurement performed using a white light interferometer (GBS, Smart-WLI), which included the correction of systematic image distortion because of nonorthogonal scanning caused by the scan mirror. After the lateral scan, stage movement in the *z*-direction was performed with a step size of $15\, \mathrm {\mu m}$. However, a refractive index mismatch between the objective lens and the medium surrounding the specimen (i.e., air and water) is detected. Therefore, the axial movement of the stage is not followed by the focal position. This difference can be expressed by a scale factor between the focal position $\Delta s_f$ and the stage displacement $\Delta z$. Considering only the marginal rays of the aperture, the following relation can be derived [21, 22]:

6 $$\begin{aligned} \Delta s_f = \bigg ( \frac{\tan (\sin ^{-1} (\textrm{NA}/n_1))}{\tan (\sin ^{-1} (\textrm{NA}/n_2))} \bigg ) \Delta z. \end{aligned}$$

The insertion of $n_1=1.0$ (air), $n_2=1.33$ (water), and $\textrm{NA}=0.42$ leads to $\Delta s_f \approx 1.39\, \Delta z$. As the detected fluorescence signal is measured based on $\Delta s_f$, $\Delta z$ is obtained by a division of 1.39. After this correction, the surface positions $z_0(x,y)$ are retrieved by least squares approximation of Eq. (3) for the different (*x*, *y*)-positions. Figure 5a shows the measurement result in the Petri dish. For comparison, the corresponding WLI reference measurement is presented in Fig. 5b. The dashed line in both images denotes the position of the surface profile extraction depicted in Fig. 5c. The machined structure can be divided into three distinct geometry elements to be resolved: the plane, the cavity, and substructures of the cavity, such as the machining marks of the manufacturing laser (see Fig. 5b). All of these elements can be identified in the fluorescence-based geometry measurement result shown in Fig. 5a. However, deviations are observed when compared with the reference measurement, which can be classified into two categories. On one hand, measurements on the left and right planes exhibit random deviations. Considering a measurement area on the flat part of approximately $0.11 \times 1.15\, \mathrm {mm^2}$ (dash-dotted rectangle in Fig. 5a), the standard deviation of the measurement amounts to approximately $7\, \mathrm {\mu m}$, while the standard deviation of the reference measurement yields $0.7\, \mathrm {\mu m}$ over the same section of the data. Accordingly, the random deviations are attributed to the measurement uncertainty of the system, which is also supported by the previous results, where an uncertainty of $8.8\, \mathrm {\mu m}$ was reported for plane surfaces [22]. On the other hand, systematic deviations occur at strong curvatures, especially an overshoot at the beginning cavity, which can be regarded as an edge artifact (cf. Fig. 5c). In these regions, the high inclinations of the surface lead to blocking of the excitation laser [23], reducing the signal-to-noise-ratio. Furthermore, an edge or high curvature can cause an asymmetrical truncation of the confocal volume, and the assumption of Eq. (1) is not valid anymore. Additionally, optical aberrations can shift or deform the resulting signal curve *S*(*z*), an effect that becomes more pronounced with increasing local surface [23]. Moreover, surface reflections are not considered by the fit function, which probably contributes to the deviations.

### Geometry Measurement Inside the LCM Etching Cell

**Fig. 6:**
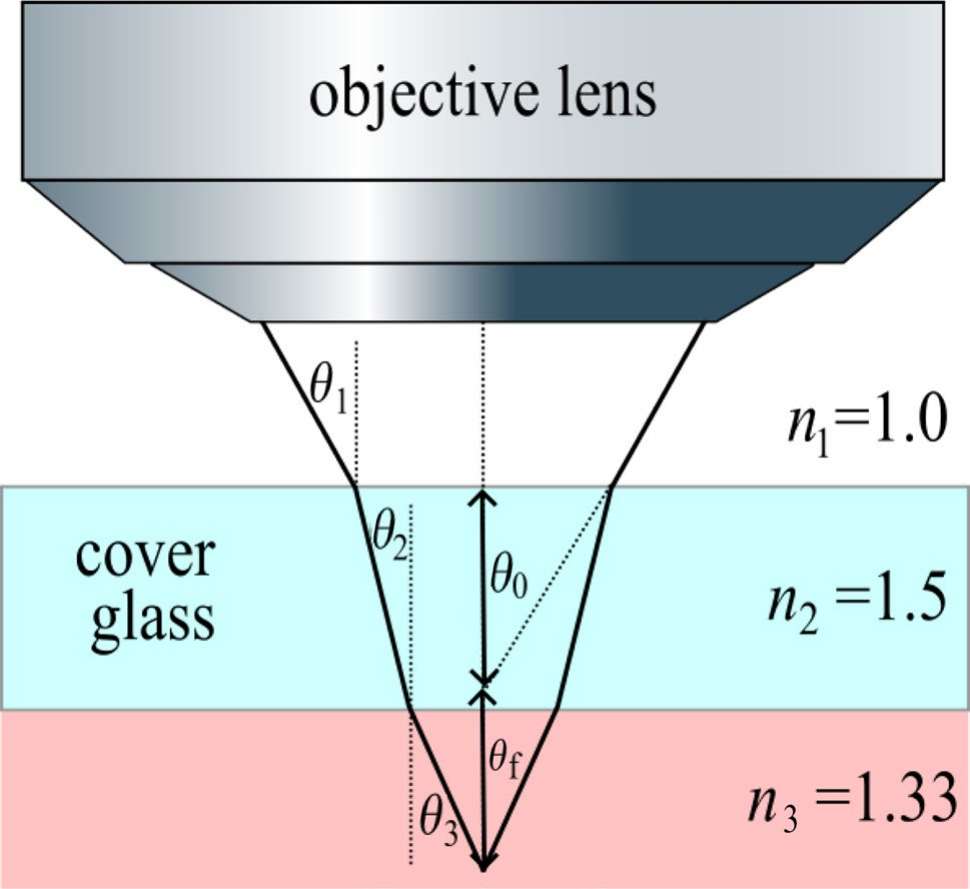
Illustration of the focal shift $s_f$ caused by refractive index mismatch at the cover glass of the etching cell and the electrolyte/fluid surface

As the next step, the geometry of the machined structure was measured inside the etching cell under fluid flow. The traversal of the cover glass of the etching cell introduces an additional refractive index mismatch, as illustrated in Fig. 6. To quantify the focal shift, Eq.(6) was modified according to the derivation in Ref. [21] to the following expression:

7 $$\begin{aligned}&\Delta s_f = \nonumber \\&\quad \bigg ( \frac{\tan (\sin ^{-1} (\textrm{NA}/n_1))}{\tan (\sin ^{-1} (\textrm{NA}/n_2))} + \frac{\tan (\sin ^{-1} (\textrm{NA}/n_1))}{\tan (\sin ^{-1} (\textrm{NA}/n_3))} \bigg ) \Delta z. \end{aligned}$$

**Fig. 7:**
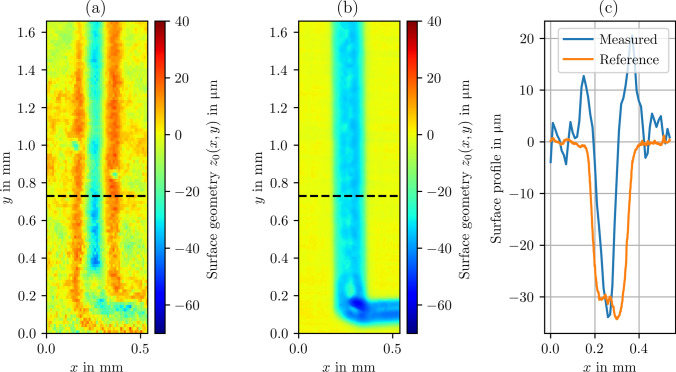
**a** Geometry measurement of the LCM structure in the etching cell with a flow velocity of $300\, \mathrm {mL/min}$, as fluid water is used. **b** WLI reference measurement. **c** Surface profiles extracted from **a** and **b** denoted by the dashed line

The insertion of $n_1=1.0$ (air), $n_2=1.5$ (glass), $n_3=1.33$ (water), and $\textrm{NA}=0.42$ leads to $\Delta z = \Delta _f/2.98$. The resulting geometry measurement is shown in Fig. 7a. Notably, a different corner of the rectangular structure was measured here. No significant effect of the fluid flow on the measurement result could be observed (after varying the flow velocity). The standard deviation of the planar region amounts to $4.6\, \mathrm {\mu m}$. Compared with that shown in Fig. 5a, the small machining marks created by the LCM infrared laser at the bottom of the cavity are no longer resolved in Fig. 7a. This loss of resolution can be attributed to the additional refraction caused by the 10-mm-thick cover glass present during the measurements, which not only results in a larger focal shift but also introduces an additional axial blur, as optical rays no longer converge as precisely at the image plane [24, 25] (Fig. 6).

### Effect of the Air Bubbles

**Fig. 8:**
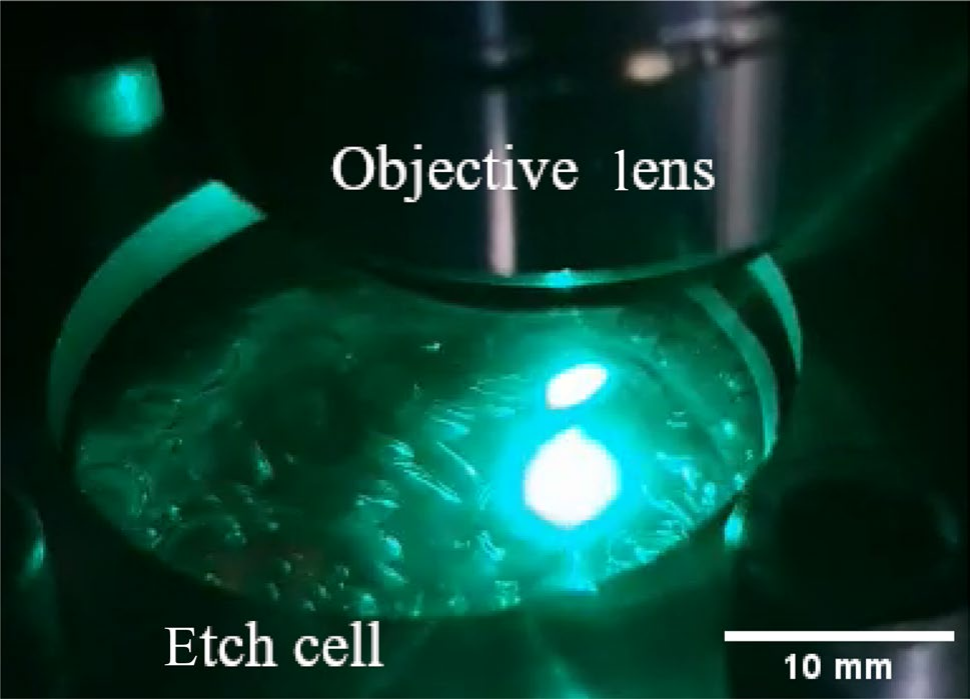
Photograph of the etching cell during the measurements while air bubbles are pumped through

**Fig. 9:**
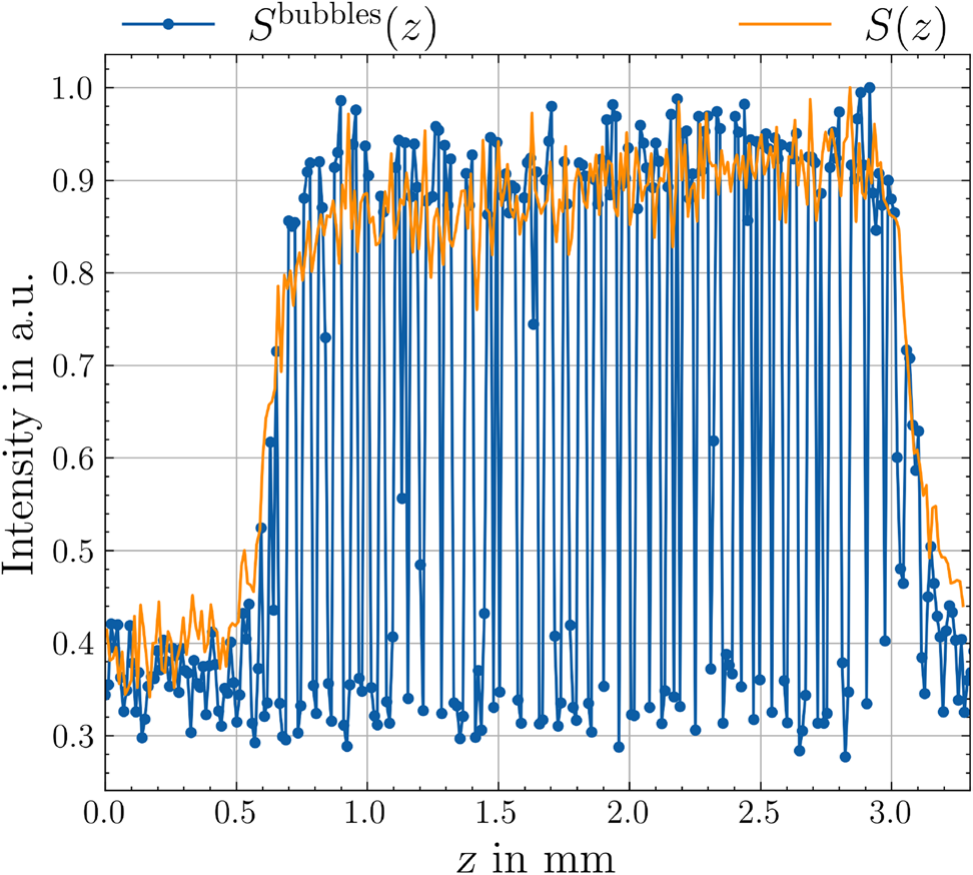
Typical measurement signal $S^{\textrm{bubbles}}(z)$ obtained for a *z*-scan at one (*x*, *y*)-position while air bubbles are pumped through the etching cell. For comparison, the measurement signal *S*(*z*) is shown without the interfering effect of the air bubbles

**Fig. 10:**
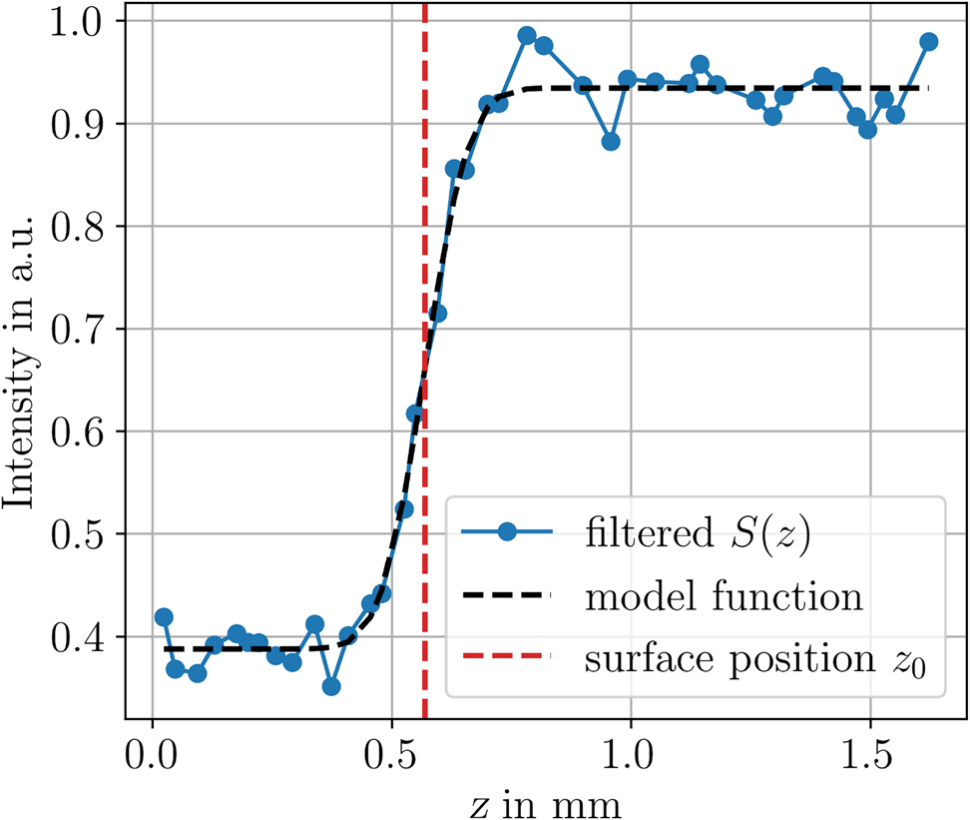
Post-processed signal data *S*(*z*) of Fig. 9 plotted along with the corresponding fit of the model function and the detected surface position $z_0$. Notably, only the first 40 data points of Fig. 9 were taken into account for the fit

**Fig. 11:**
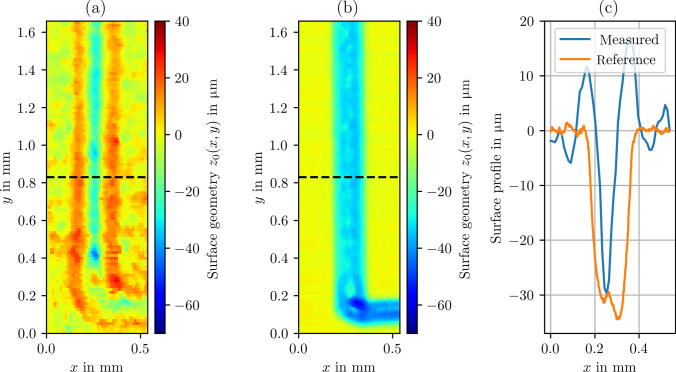
Measurement result of the LCM structure **a** in-situ with air bubbles present in the optical path and **b** by WLI (reference). As process fluid, Rhodamine B (RhB) dissolved in water was used. **c** Surface profiles extracted from **a** and **b** denoted by the dashed line

To test the robustness of the indirect approach for the surface geometry measurement against the occurrence of highly dynamic boiling bubbles, the pump was operated in a way that allowed air to be drawn in. As a result, the solution of water with RhB contains air bubbles. To ensure a relatively constant stream of air bubbles, the flow velocity was increased to $800\, \mathrm {mL/min}$. Figure 8 shows a photograph of the streaming air bubbles in the etching cell, illustrating the turbulent flow conditions and the size of the air bubbles. The impact of these conditions on the measurement signal is shown in Fig. 9. As a result, the signal data show a strongly oscillating character. To evaluate these noisy data, an evaluation approach similar to that reported in Ref. [26] was chosen. As the first postprocessing step, all local maxima of the data were detected, and the remaining data were discarded. Because some outliers were still present in the processed data, a moving median with a window size of 3 data points was computed and subtracted from the data. On the resulting dataset, a one-sided Smirnov–Grubbs test (min) was applied to eliminate the outliers [27]. Then, a Gaussian error function $S(z) = a\cdot \textrm{erf} (z -z_0)\cdot b +c$ with parameters *a*, *b*, *c* was fitted to the first 40 data points of the remaining dataset, see Fig. 10. A few outliers resulting from the fit were eliminated by a hot or cold pixel filter, i.e., replaced with the median of the neighboring data points. The final measurement result of the geometry is shown in Fig. 11. Despite the strong interference of the signal, the geometry measurement result is comparable to those shown in Fig. 7a, indicating the robustness of the measurement principle against streaming air bubbles. The standard deviation of the planar region amounts to $4.2\, \mathrm {\mu m}$. However, the measurement is not entirely unaffected, i.e., the cavity appears narrower in this case, and the overshooting edge artifacts are slightly distributed.

### Temperature Measurement in the LCM Environment

To evaluate the feasibility of fluorescence-based temperature measurement in the LCM process environment, the phosphoric acid pumped through the etching cell was heated up gradually, regulated by a thermostat. The flow velocity was $300\, \mathrm {mL/min}$. Assuming a uniform fluid temperature, the focal spot of the excitation laser was positioned at the center of the fluid. The spatial assignment of the fluorescence decay was not examined in this study. Therefore, the pinhole was removed. Notably, the instrument response function was not considered. The fluorescence decay was measured across electrolyte temperatures ranging from approximately 20–95 °C, with 10 decay measurements taken for each temperature.

**Fig. 12:**
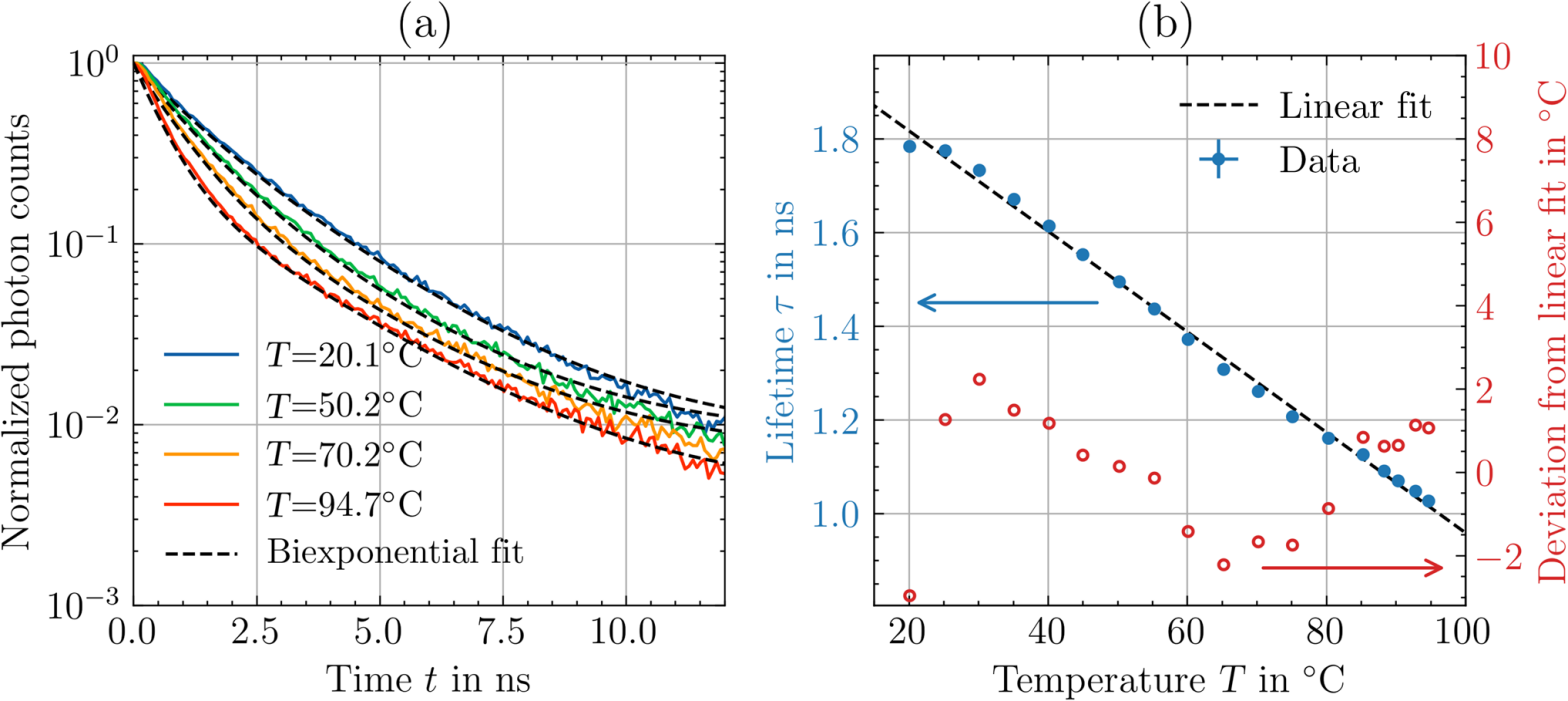
**a** Exemplary fluorescence decay curves for four different temperatures with RhB dissolved in phosphoric acid. **b** Resulting calibration curve of the fluorescence lifetime $\tau$ as a function of the temperature *T* (averaged over 10 measurements) plotted along with the deviation of the temperature *T* from the linear fit

Figure 12a shows exemplary fluorescence decay curves measured for different temperatures. In addition, the biexponential function is shown as a dashed curve for each measurement, which results from curve fitting of Eq. (4) to the data. As a result, the parameters *a*, $\tau _1$, and $\tau _2$ and an additionally considered offset to account for the background signal are determined from the fit. The fit range extends from the decay peak to $12\, \textrm{ns}$ after the decay peak. The average lifetime $\tau$ was calculated for each measured decay curve using Eq. (5).

The dependence of the resulting lifetime $\tau$ from the temperature *T* (averaged over 10 measurements per temperature) is shown in Fig. 12b. The minimal and maximal standard deviations indicated by error bars are 0.005 and $0.013\, \textrm{ns}$, respectively. The mean value of the standard uncertainty amounts to $0.007\, \textrm{ns}$. The resulting experimental data indicate a linear relationship between the temperature *T* and the lifetime $\tau$. Thus, the following linear equation of the temperature *T* (calibration curve) is obtained:

8 $$\begin{aligned} T (\tau ) = -93(2) ^{\circ }\hbox {C}\, \mathrm {ns^{-1}} \cdot \tau + 189(2)^{\circ }\hbox {C}. \end{aligned}$$

For a change in temperature *T* of $10\,^{\circ }\hbox {C}$, the change in lifetime $\tau$ is approximately $0.1\, \textrm{ns}$. Therefore, the standard deviation of a single temperature measurement is in the order of $1\,^{\circ }\hbox {C}$ (random error). The root-mean-square rms of the deviations between the linear calibration curve and the experimental data yields ${\text{rms}} = 1.4\,^{\circ }\hbox {C}$ (systematic error). As a result, the feasibility of precise and accurate fluorescence-based temperature measurements in the LCM environment is proven.

## Conclusion

This study presents an in-situ-capable fluorescence-based measurement approach for the workpiece geometry and fluid temperature for LCM. The setup employs a confocal fluorescence microscope and a 3D scanning system to capture the fluorescent light emitted by RhB dissolved in the flowing process fluid. The temperature was determined by measuring the fluorescence lifetime via the well-established TCSPC method. For geometry measurements, an optical signal model was applied to determine the surface position indirectly from the fluorescence intensity gradient along the *z*-axis. Notably, although the feasibility of each measurement approach was analyzed separately, the geometry and the temperature measurement system already use an identical optical path that was realized as an on-machine solution. Only photodetection was realized with different detectors. To evaluate the feasibility of geometry measurements, a structure produced by LCM was measured and compared with the WLI reference measurements. Furthermore, for a detailed analysis of the surface topography, representative surface profiles were extracted. Thereby, the influence of the etching cell, fluid flow, and streaming air bubbles on the geometry measurements was investigated. The results showed that the proposed measurement approach successfully provides an areal topology map of the machined structure within the etching cell containing streaming fluid, even in the presence of air bubbles (simulating boiling bubbles). The measurement uncertainty for planar regions amounts to $7\, \mathrm {\mu m}$, whereas additional systematic deviations occurred at the edges of the machined cavity. These non-negligible measurement deviations are presumably caused by an asymmetrical truncation of the confocal volume and scattering effects or light reflections on the surface, which are not accounted for in the current signal model. Additionally, optical aberrations likely contribute to these systematic deviations. Therefore, enhancement of the signal model and data evaluation should be investigated in future work. Notably, the focus here was on the application of the indirect measurement principle together with confocal fluorescence microscopy in the practical environment of LCM. The measurement setup was initially designed to ensure the largest possible working distance from the etching cell. In the future, the etching cell could also be optimized so that an objective with a higher numerical aperture and smaller working distance could be used, which could significantly improve the uncertainty and measurement resolution. Furthermore, the fluorescence lifetime method can access the temperature of the electrolyte solution precisely with a random error of $1\,^{\circ }\hbox {C}$ and a systematic error of $1.4\,^{\circ }\hbox {C}$ within the LCM environment. The combination of this measurement with an axial scan has significant potential to simultaneously measure both temperature and geometry using the single-photon counting detector. Future work will focus on increasing the resolution and precision of the proposed geometry measurement for the LCM process. To achieve this goal, the use of a high-resolution objective lens, higher sampling rates, and an improved signal evaluation algorithm is planned.

## Data Availability

The data will be made available on reasonable request.

## References

[1] Shao Y, Sun S-F, Liu G-L, Wang P-P, Shao J, Zhang F-Y, Wang X (2021) Laser-assisted thermochemical ultrahigh-precision polishing of titanium in phosphoric acid solution. Int J Adv Manuf Technol 115(4):1201–1210. 10.1007/s00170-021-07267-0

[2] Messaoudi H, Mikulewitsch M, Brand D, Freyberg A, Fischer A (2019) Removal behavior and output quality for laser chemical machining of tool steels. Manuf Rev 6:13. 10.1051/mfreview/2019015

[3] Bouraoui Y, Rathmann L, Niehaves C, Mikulewitsch M, Fischer A, Radel T (2024) Material removal in laser chemical processing with modulated laser power. J Laser Appl 36(1):012013. 10.2351/7.0001109

[4] Mehrafsun S, Vollertsen F (2013) Disturbance of material removal in laser-chemical machining by emerging gas. CIRP Ann 62(1):195–198. 10.1016/j.cirp.2013.03.030

[5] Lu Y, Bouraoui Y, Niehaves C, Fischer A, Radel T (2024) Laser power modulation between disturbed and undisturbed material removal regime in laser chemical processing. Mendeley Data. 10.17632/K66NRTPNN5.1

[6] Simons M, Radel T, Vollertsen F (2021) Extension of the process window in laser chemical machining by temperature-dependent reduction of the electrolyte viscosity. Int J Precis Eng Manuf 22(8):1461–1467. 10.1007/s12541-021-00548-4

[7] Loeschner U, Schille J, Streek A, Knebel T, Hartwig L, Hillmann R, Endisch C (2015) High-rate laser microprocessing using a polygon scanner system. J Laser Appl 27(S2):29303. 10.2351/1.4906473

[8] Leggio L, Di Maio Y, Pascale-Hamri A, Egaud G, Reynaud S, Sedao X, Mauclair C (2023) Ultrafast laser patterning of metals commonly used in medical industry: surface roughness control with energy gradient pulse sequences. Micromachines 14(2):251. 10.3390/mi14020251 36837953 10.3390/mi14020251 PMC9967074

[9] Liu J, Liu C, Tan J, Yang B, Wilson T (2016) Super-aperture metrology: Overcoming a fundamental limit in imaging smooth highly curved surfaces. J Microsc 261(3):300–306. 10.1111/jmi.12334 26565890 10.1111/jmi.12334

[10] Fischer A, Mikulewitsch M, Stöbener D (2020) Indirect fluorescence-based in situ geometry measurement for laser chemical machining. CIRP Ann 69(1):481–484. 10.1016/j.cirp.2020.03.018

[11] Maruno K, Michihata M, Mizutani Y, Takaya Y (2016) Fundamental study on novel on-machine measurement method of a cutting tool edge profile with a fluorescent confocal microscopy. Int J Autom Technol 10(1):106–113. 10.20965/ijat.2016.p0106

[12] Takaya Y, Maruno K, Michihata M, Mizutani Y (2016) Measurement of a tool wear profile using confocal fluorescence microscopy of the cutting fluid layer. CIRP Ann 65(1):467–470. 10.1016/j.cirp.2016.04.014

[13] Yoshikawa M, Fujii S, Kadoya S, Sugihara T, Michihata M, Takahashi S (2024) Fluorescence-Based calibration model for in-situ measurement of micro-scaled lubricant thickness distribution at indentation interface. Nanomanuf Metrol 7(1):13. 10.1007/s41871-024-00232-x

[14] Migler KB, Bur AJ (1998) Fluorescence based measurement of temperature profiles during polymer processing. Polym Eng Sci 38(1):213–221. 10.1002/pen.10182

[15] Lakowicz JR (2016) Principles of Fluorescence Spectroscopy, 3rd edn. Springer, New York

[16] Becker W (2012) Fluorescence lifetime imaging - techniques and applications. J Microsc 247(2):119–136. 10.1111/j.1365-2818.2012.03618.x 22621335 10.1111/j.1365-2818.2012.03618.x

[17] Okabe K, Inada N, Gota C, Harada Y, Funatsu T, Uchiyama S (2012) Intracellular temperature mapping with a fluorescent polymeric thermometer and fluorescence lifetime imaging microscopy. Nat Commun 3(1):705. 10.1038/ncomms1714 22426226 10.1038/ncomms1714 PMC3293419

[18] Tsuji T, Yoshida S, Yoshida A, Uchiyama S (2013) Cationic fluorescent polymeric thermometers with the ability to enter yeast and mammalian cells for practical intracellular temperature measurements. Anal Chem 85(20):9815–9823. 10.1021/ac402128f 24047471 10.1021/ac402128f

[19] Mehdi S, Yangpeng L, Hadrien C, Fabrice L, Xishi W, Guillaume C (2021) Fluorescence lifetime measurements applied to the characterization of the droplet temperature in sprays. Exp Fluids 62(8):174. 10.1007/s00348-021-03264-x

[20] Rüttinger S, Buschmann V, Krämer B, Erdmann R, Macdonald R, Koberling F (2008) Comparison and accuracy of methods to determine the confocal volume for quantitative fluorescence correlation spectroscopy. J Microsc 232(2):343–352. 10.1111/j.1365-2818.2008.02105.x 19017233 10.1111/j.1365-2818.2008.02105.x

[21] Visser T, Oud J, Brakenhoff G (1992) Refractive index and axial distance measurements in 3-D microscopy. Optik 90(1):17–19

[22] Mikulewitsch M, Auerswald MM, Freyberg A, Fischer A (2018) Geometry measurement of submerged metallic micro-parts using confocal fluorescence microscopy. Nanomanuf Metrol 1(3):171–179. 10.1007/s41871-018-0019-6

[23] Zhang Y, Gu K, Li Y, Liu J, You X, Wang Y (2024) Influences of fluorescence film thickness and optical aberrations on curved surface metrology in fluorophore-aided scattering confocal microscopy. Opt Commun 550:129960

[24] Müller M (2006) Introduction to Confocal Fluorescence Microscopy, vol 69, 2nd edn. Tutorial texts in optical engineering. SPIE Press, Bellingham

[25] Diel EE, Lichtman JW, Richardson DS (2020) Tutorial: Avoiding and correcting sample-induced spherical aberration artifacts in 3D fluorescence microscopy. Nat Protoc 15(9):2773–2784. 10.1038/s41596-020-0360-2 32737465 10.1038/s41596-020-0360-2

[26] Feld B, Gebken J, Tausendfreund A, Fischer A (2024) Indirect optical geometry measurements with a stream of particles as micro probes. Opt Lasers Eng 183:108539. 10.1016/j.optlaseng.2024.108539

[27] Grubbs FE (1969) Procedures for detecting outlying observations in samples. Technometrics 11(1):1–21. 10.1080/00401706.1969.10490657

